# Eolian chronology reveals causal links between tectonics, climate, and erg generation

**DOI:** 10.1038/s41467-022-33316-7

**Published:** 2022-09-29

**Authors:** Shlomy Vainer, Ari Matmon, Yoav Ben Dor, Eric P. Verrecchia, Frank Eckardt, ASTER Team, Georges Aumaître, Didier L. Bourlès, Karim Keddadouche

**Affiliations:** 1 Institute of Earth Sciences, The Hebrew University of Jerusalem, 919040 Jerusalem, Israel; 2 Institute of Earth Surface Dynamics, University of Lausanne, 1015 Lausanne, Switzerland; 3 Aix-Marseille Université, CNRS, Collège de France, IRD, INRA, CEREGE, 13545 Aix-en-Provence, France; 4 Geological Survey of Israel, 32 Yesha’ayahu Leibowitz, 9692100 Jerusalem, Israel; 5 Dept. Environ. & Geog. Sci., University of Cape Town, Cape Town, 7701 South Africa

**Keywords:** Climate and Earth system modelling, Environmental impact, Geomorphology, Sedimentology

## Abstract

Evaluating the impact and implications of eolian repositories that mark large-scale climatic transitions requires knowledge about the timing of their emplacement and the mechanisms responsible for their production, which remain highly uncertain. Here we apply numerical modeling of cosmogenic nuclide data, measured in the largest continuous terrestrial body of sand on Earth, to determine settings under which the sand was generated, by constraining the timing of sand introduction into the interior of southern Africa. Our findings reveal that major events of sand formation and accumulation in the Kalahari Basin occurred between ~2.2 and 1 Myr ago. The establishment of the Kalahari sand field corresponds to regional, continental, and global scale morphotectonic and climatic changes that contributed to the mass production and widespread dispersion of sand. These changes substantially altered existing habitats, thus constituting a crucial milestone for flora, fauna, and hominins in southern Africa during the Pleistocene.

## Abstract (web summary)

Modeling cosmogenic nuclides concentrations from Kalahari Desert Sand reveals the time of sand introduction into the landscape. This coincides with morphotectonic and climatic changes that could have triggered sand production and its impact on the environment.

## Introduction

**Fig. 1:**
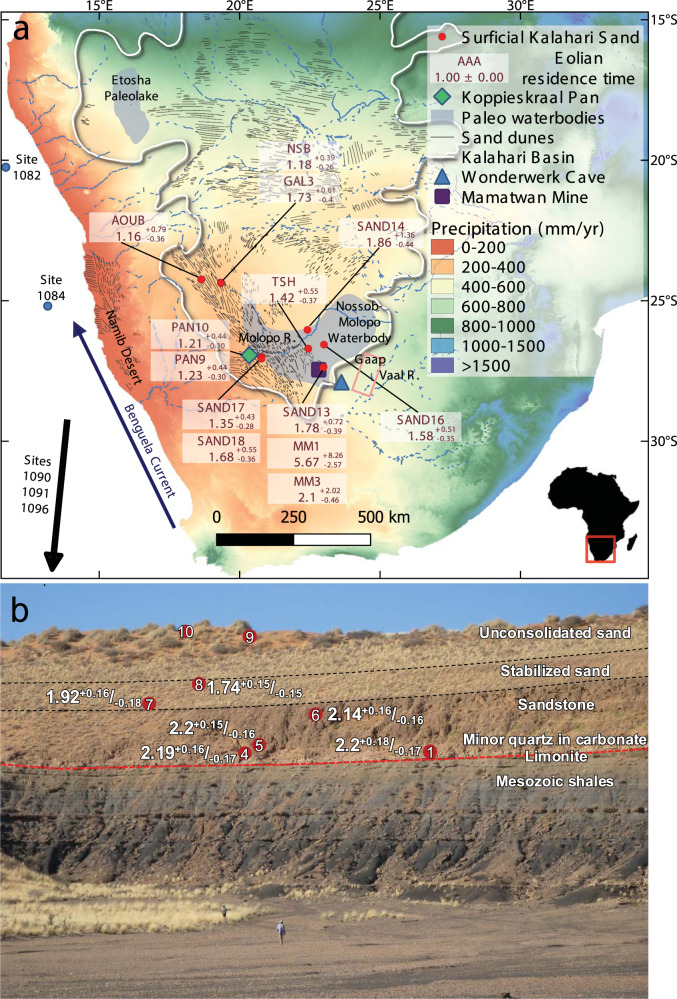
Eolian chronology of the Kalahari Desert Sand. **a** Locations of sand samples and sites mentioned in the text are superimposed on the modern precipitation map of southern Africa (map made with QGIS. Climate data is from Fick, S.E. and R.J. Hijmans. WorldClim 2: new 1 km spatial resolution climate surfaces for global land areas. International Journal of Climatology 37 (12): 4302–4315, 2017). Eolian residence time is the weighted average of the simulated duration in which agreement between simulated and measured radionuclides was achieved and is marked with a black line in Fig. 2. Inset: the red rectangle depicts the area of the main figure within the African continent (image from Vecteezy.com). **b** A 60 m thick exposure at the northeastern wall of Koppieskraal Pan (see location in Fig. 1a) where ~40 m of consolidated Kalahari Sand overlies Mesozoic shales of the Karoo supergroup. Burial ages (reported in Ma with 1σ uncertainty) were calculated assuming pre-burial ^*26*^*Al/*^*10*^*Be* between 5.56 and 5.79 (estimated iteratively after^28^). Numbers in red circles correspond to the sample’s serial number in the supplementary data.

Eolian sand deposits cover ~20% of the world’s drylands and have been shown to be linked with and record substantial changes in terrestrial environments^1^. The instigation of eolian activity in the largest deserts of the world was suggested to mark either the onset or significant increase in continental-scale aridity^2–4^. However, the simplified associations between eolian activity and aridity have been questioned e.g., ref. 5. Several factors are responsible for this relationship, including (1) wind energy and its direction, the type and distribution of vegetation and biogenic crust covers, and sediment generation and supply, all of which govern eolian dynamics and have contrasting effects, and (2) the tendency of aeolian systems to exist under different conditions demonstrated by the non-coherent basin-scale chronological correlations of both eolian and non-eolian proxies^5–9^. A multitude of studies have addressed the controversial relationship between dune activity and prevailing environmental conditions within the Kalahari during the late Quaternary e.g., ref. 5, particularly in relation to the reworking and redeposition of sediments that can be dated by Optically Stimulated Luminescence (OSL). However, beyond the limits of OSL dating (c. 300ka in the Kalahari) little is known about when or how the deep sand mantle of the Kalahari basin was generated. Here we attempt to address this issue using cosmogenic nuclides techniques by establishing (1) the eolian residence time (i.e. the time since sediment became available for eolian transport) while performing a simplified model of sand erosion and fluvial transport followed by eolian transport in mobile dunes and (2) the initial emplacement of that sediment into the Kalahari through burial dating of consolidated sand. In this work, we reconstruct the million-year scale chronological framework of eolian sand transport (considering boundary conditions set by bedrock erosion and fluvial transport and subsequent various eolian pathways) and emplacement. The extensive sand cover of the Kalahari Basin (Fig. 1a) is the largest body of sand on Earth, and it provides the major regional repository for Quaternary environmental conditions in southern Africa. The arid sector of the Kalahari Desert is used as a case study to examine causal links between periods of eolian activity^5^ and the contemporaneous environmental settings.

The southern part of the African continent lies within a pronounced rainfall gradient (Fig. 1a) where alongside the descending limb of the Hadley Cell, the regional atmospheric circulation is governed by the Intertropical Convergence Zone and associated Tropical Rain Belt, the Congo Air Boundary, and temperate frontal systems within the southern hemisphere westerlies in the west and south. Moisture influx into the sub-continent is primarily driven from the Indian Ocean via easterly winds and is inhibited along its western coastline, where moisture depleted atmospheric patterns are reflected in the development of the hyper-aridity of the Namib Desert. The marine record of the Southeast Atlantic that preserves the chronology of Southern Hemisphere glaciation and the development of the Benguela Current provides a general timeframe of key climate fluctuations on the western continental margins^10^. At the eastern African terrestrial margins, where climate has developed along a different trajectory than the continental interior^11^, there are some rift-filling lacustrine archives that preserve long enough continuous records to identify climate changes^12,13^.

Tectonic activity in southern Africa is not uncommon, despite the intracratonic settings that accommodate the Kalahari. This is primarily due to the extension of the East African Rift System, which terminates in the interior of the subcontinent within the middle of the Kalahari Basin^14^, and the epeirogenic vertical movements within the basin itself and along its margins^15^. Pleistocene activity in the central Kalahari is reflected by faulting of the stabilized Kalahari Sand (main sedimentary fill of the basin)^16,17^ and intraplate seismicity, which has been documented at the southern tip of the continent^18^. This tectonism is related to the intraplate Wegener stress anomaly which extends over 15° of latitude to the north, and creates regional, coast-parallel, horizontal stresses, due to rotation between the Somalia and Africa plate and possibly the presence of the southern African superplume^19^.

Direct dating of eolian sand deposits at the million-year timescale in the Kalahari is hampered by the lack of suitable sites and chronometers^20–22^. This had resulted in a controversy concerning the origin of regional sand deposits, which in turn led to substantially varied estimations of their initial production and subsequent distribution, ranging over tens of millions of years^23–25^. While many OSL ages were published for the Kalahari dune fields^26^, the fact that OSL signals reset upon exposure of the sand to light, means that these OSL ages only provide the last phases of dune migration, and never exceed 300Ka in the Kalahari due to signal saturation^5,22^. Overall, the geomorphological response to environmental changes that occurred during the early Pleistocene in the main continental portion of southern Africa remains poorly dated^5,14,24^.

To determine when sand was detached from its parent bedrock and to unravel the phases of sand turnover by the eolian movement since it first became available to transport, we measured the concentrations of ^26^Al and ^10^Be in two sets of samples from the Kalahari Desert. Each set was modeled by a different approach. (Fig. 1a, b): (1) thirteen samples of surficial sand were collected along a 600 km transect, and (2) six samples were collected along a depth profile of buried sandstones in the southwestern Kalahari (Koppieskraal Pan). Eolian transport of the samples was established through their grain size characteristics (Supplementary Data 1). The surficial sand samples were numerically modeled by simulating their mobilization using the Cosmolian program that generates simulations of eolian movement^27^. The simulations are dependent on previously reported OSL ages of eolian and fluvial sediments (see Methods section), and on the concentrations of ^26^Al and ^10^Be which accumulated during repeated sand burial during transport. The buried sandstone samples were analyzed by employing cosmogenic nuclide burial dating to determine when the sand was deposited^21,28,29^.

In the present work, we compare our results with independently established proxies and published timeframes of regional and global climatic and environmental shifts and identify possible triggers causing eolian vitality or dormancy. Furthermore, we point to the specific combination of conditions that would facilitate the formation and expansion of eolian realms as well as the implications of massive sand deposits on landforms and habitats.

## Results and discussion

### The timing of sand introduction to the Kalahari

**Fig. 2:**
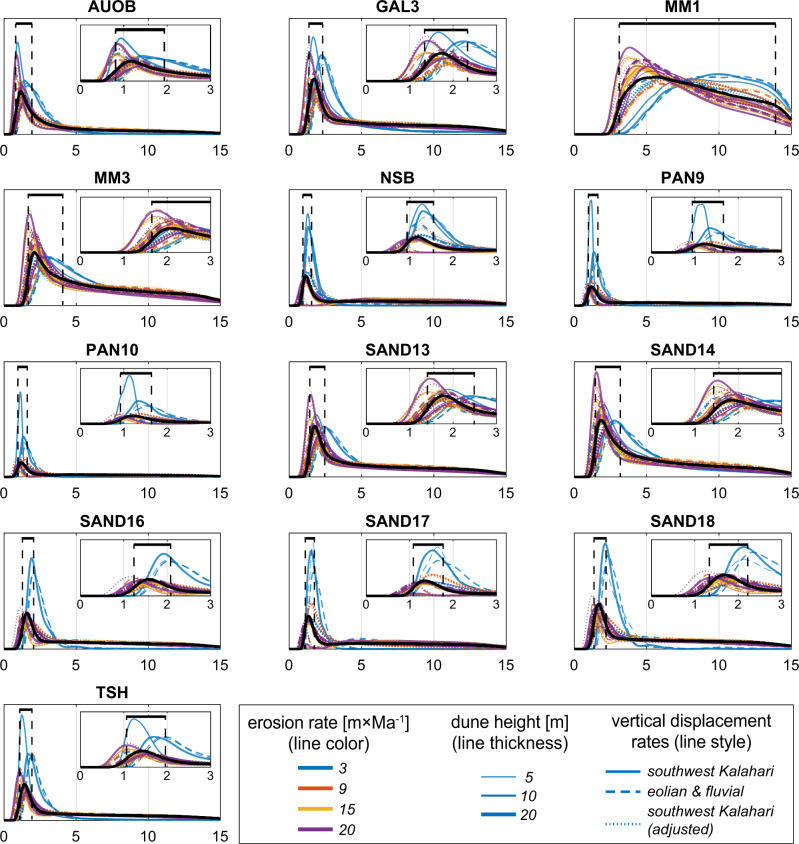
Kernel density estimates of simulations performed to assess eolian residence time of Kalahari Sand. Simulations were carried out using the Cosmolian program^27^ in which modeled concentrations of ^*26*^*Al* ^*10*^*Be* simulated measured values in Kalahari Sand samples. Results are the outcome of 10,000 simulations for each of the 36 combinations of parameters representing possible scenarios considered for each sample. These boundary conditions include four paleo-erosion rates, three dune heights, and three datasets of vertical displacement rates constructed from luminescence and ^14^C ages of the Kalahari Sand. In twelve out of thirteen samples, kernel density estimates are clustered into distinct peaks that range within a narrow time span. For each sample, the weighted average of all simulations is shown with a black solid line, and its highest probability is interpreted as the eolian residence time. The uncertainty of the black line was calculated with full-width at half-maximum approach, and is marked with dashed black lines. The name of each sample corresponds to the names in Fig. 1a.

The eolian residence time (see Methods section) of surficial sand is clustered into a distinct timespan, implying that sands in the studied sites in the Kalahari Desert were eroded from their parent bedrocks and started to accumulate between ~2.1 and 1.2 Ma (Fig. 1). Kernel density estimates of the simulations that were carried out considering bedrock erosion rates of 9, 15, and 20 m∙Ma^−1^ are similar. However, simulations carried out using a lower erosion rate of 3 m∙Ma^−1^ (representing the currently arid climate), deviate from the general pattern constructed by the rest of the scenarios (Fig. 2). If sand had arrived from areas characterized by higher erosion rates than today (see Methods section), it could have been due to higher elevation and greater relief or more humid climatic conditions at the source during the time of sand disengagement^30,31^. Burial ages signify that deposition at Koppieskraal Pan (Fig. 1b) took place within a narrow timespan, between 2.2 and 1.7 Ma (Fig. 1b). This is in agreement with the independently determined timeframe for the eolian deposition of the Kalahari Sand: it started at 4 ± 1 Ma, as was estimated from the youngest fossils of the desiccated Etosha Paleolake that underlie Kalahari Sand^32^, and ended by ~1 Ma, at a time when fluvial deposits that underlie eolian sand in Mamatwan Mine were buried by the sand (Fig. 1a)^21^. It further agrees with the existence of red Kalahari sand below artifact-bearing layers of the Rietputs gravels in the Vaal River that are dated using cosmogenic nuclides to 1.4–1.8 Ma^33,34^.

Furthermore, our results compare well with other regional studies that indicate shifting environmental conditions during the late Pliocene-early Pleistocene. An inland repository that archives key shifts in climatic and environmental conditions prevails in the form of near-shore sediments of the Namib Desert^29,35^. Farther inland, sparse palaeoclimatological data for the early Pleistocene has been derived from the southern Kalahari margins^14^. There, a period of protracted erosion was inferred to predate the development of the desert, based on spring and tufa deposits developed on the dolomites of the Gaap Escarpment (Fig. 1a). ^36^ applied hominin fossils and tool-based chronological constraints to draw a paleoclimatic sequence extending to the early Pleistocene, or possibly earlier. In their study, the enduring period of erosion is represented by a layer containing sand and baboon remains. These remains have the most in common with member 1 in the Swartkrans Cave (2.22 ± 0.09 Ma^37^), and with member 5 in Sterkfontein Cave (dated to 2.18 ± 0.0.21 Ma^38^). This erosional phase could have started as early as 3.49 ± 0.19, which is the age of Australopithecus Africanus at Sterkfontein Cave^39^, that correlates with Australopithecus fossil that was recovered with the baboons at the Gaap Escarpment. On the western edge of the Gaap Escarpment, principal component analysis of phytolith data alongside faunal assemblages and stable carbon and oxygen isotope data measured in enamel finds in the Wonderwerk Cave (Fig. 1a), lead to the conclusion that conditions, wetter than today, existed throughout the early Pleistocene. These wetter conditions terminated at ca. 0.9 Ma^40^. Within the same sub-basin, a provenance study that was performed on a 55 m section of Quaternary, or earlier sediments^21,41^, and that utilized geochemical and sedimentological properties to reconstruct the environmental conditions during their deposition, had reached a similar conclusion of a pronounced geomorphic response to a change at around 1 Ma, leading to lowering of regional water tables^30^. Finally, a similar transition was suggested based on field observations performed in the well-integrated drainage network of the Molopo River (Fig. 1a), which became defunct due to the massive infiltration of the Kalahari sand^24^. However, this event was poorly constrained in time.^24^ suggested, without direct evidence, that this transition occurred following the changes in northern Hemisphere climatic cycles, at the late Pliocene, and with the faunal changes that were described in the Sterkfontein Cave, and correlated with member 4 (i.e. <3.67 ± 0.16 Myr^38^).

### The presentation of sand and contemporaneous environmental settings

**Fig. 3:**
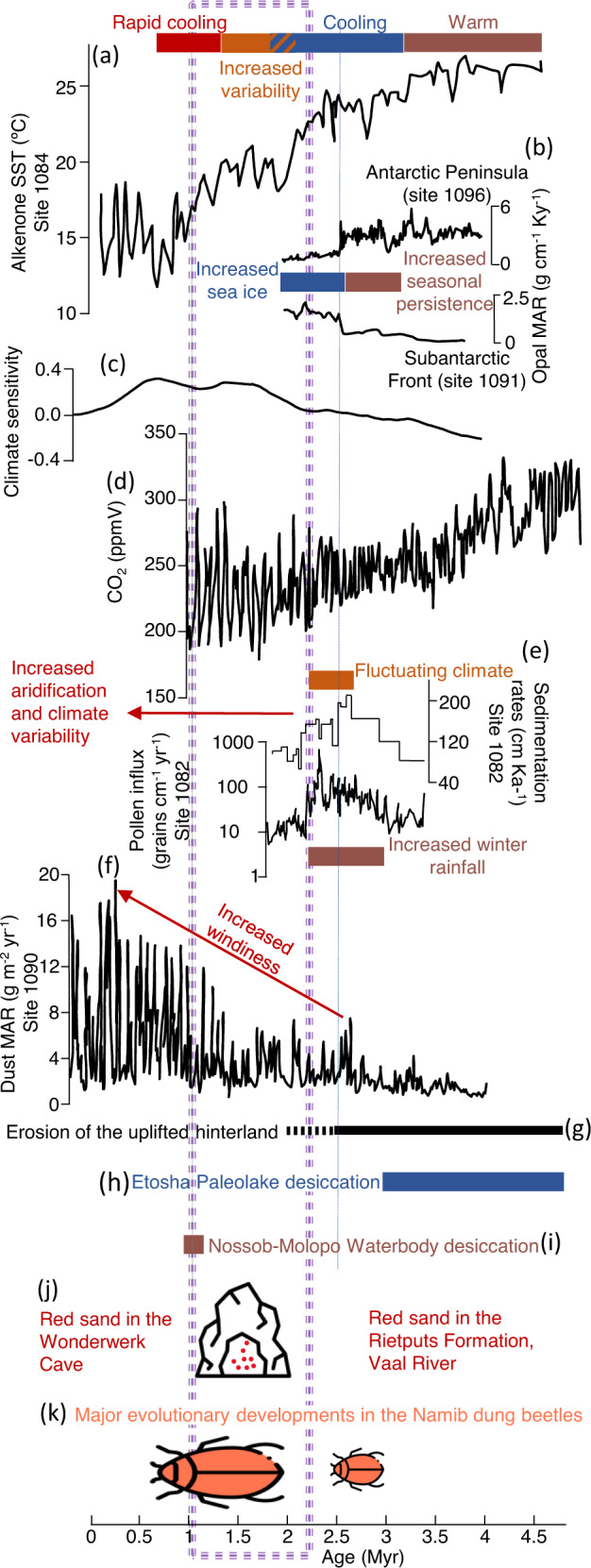
Environmental framework for the generation of the Kalahari Erg. The timing of initial sand introduction in the Kalahari Desert (purple dashed rectangle) is displayed on top of environmental settings and key events in southern Africa and proximate oceans. The text provides interpretation from previous studies of the records and is colored in accordance with colored bars and arrows. **a** Sea Surface Temperature (SST) record offshore Namibia (ODP site 1084) derived from alkenone unsaturated index (${U}_{37}^{K{\prime} }$), which reflects the strengthening of upwelling along the west African margins throughout the Pleistocene^10^. **b** Primary productivity based on biogenic opal Mass Accumulation Rates (MAR) shows a coeval increase in the Southern Ocean (ODP site 1091) and decrease in the Antarctic Peninsula (ODP site 1096) due to the onset of Antarctic Sea Ice^49^. **c** Climate sensitivity in high latitudes, calculated as the ratio between the deconvoluted records of the obliquity component of the scaled oxygen isotope and the solar forcing^47^. **d** Reconstructed atmospheric CO_2_ record showing a decrease below the bioclimatic threshold for the expansion of C_4_ plants at ~2.7 Ma^54^. **e** Sedimentation rates and pollen Influx at ODP Site 1082 indicate a transition from a mixed fluvial and eolian input to predominantly eolian transport at 2.2 Ma^57^. **f** Dust MAR at site 1090 derived from Ti MAR recording increased windiness since ~2.7 Ma^48^. **g** The southern Kalahari is breached by the erosive power of the Molopo following uplift in the range of hundreds of meters^24^. **h** The vast northern Kalahari Etosha paleolake is filled with sand^32^. **i** The Nossob-Molopo Waterbody is filled with gravel^30^. **j** The Wonderwerk Cave in the southern Kalahari rim is filled with sand as well as red sand is found in the Rietputs Formation in the Vaal River^33,34,59^. (k) Risen of new subgenus due to environmental stress in the Namib ~2 Ma^63^. Beetle and cave icons were made by Freepik from www.flaticon.com.

Our results coincide with several events that could have impacted the production and transport of sand (Fig. 3). During the end of the Pliocene and the Early Pleistocene, the landscape probably responded to vertical crustal movements that could have caused substantial uplift along several structural axes in southern Africa^30,41–43^. Although not all researchers agree with the idea of significant uplift^44^, such uplift could have triggered the creation of the Kalahari Erg^45^. Indeed, this uplift would have favored the production of detritus through the dissection of the deformed elevated surfaces, while erosion was enhanced by chemical weathering due to the warmer south Atlantic Ocean waters^10^. Accordingly, flattening due to weathering and erosion, characterizes the elevated topography^45^. The uplifted terrains altered the climatic conditions in the continental interior by blocking ocean-derived moisture, thus promoting sand distribution. This regional effect was amplified by transitions in global climate dynamics^46^, responding to high latitude solar forcing^47^ as inferred from δ^18^O in benthic foraminifera (site 1090^48^). A pronounced climatic shift is recorded at ODP sites 1091 and 1096, which are located 2000–3000 km south of our study sites, where increased cooling and changes in heat transport are observed between 3.2–2.6 Ma^49^, as well as increased dust deposition at 2.7 Ma (site 1090^48^). Indeed, a multi-proxy compilation of records offshore the Namib Desert (site 1084) demonstrates that the cooling trend at the south Atlantic propagated northwards between 3.2 and 1.0 Ma^10^.

The climatic deterioration escalated between 1.9 and 1.7 Ma, as indicated by the variations in biogenic silica assemblages in the Southern Ocean^50^. During this period, eolian deposition of sand took place at Koppieskraal Pan, while a second peak of dust flux in the Southern Ocean accumulated (site 1090^48^). The developed orography and climate fluctuations that characterized southern Africa during the late Pliocene and the early Pleistocene were essential for establishing the Kalahari Erg^45^. These enabled: (1) accelerated denudation and canyon incision^51^, and (2) augmented long-distance transport of eolian silt^52^. The interplay between successive humid and arid conditions taking place within a bimodal topography would have resulted in weathering of the elevated margins followed by eolian propagation of the sediments by wind within the subsidizing basin^53^.

Sand formation was further encouraged by the sharp decrease in CO_2_ levels that began around 3 Ma^54^, stimulating sand dispersion by increasing the density of C_4_ grass cover during the Pleistocene, when concentrations fell below the 250 ppm threshold^11^. This in turn would have facilitated the mobilization of the freshly produced sand, by reducing the tree to grass ratio. Such changes in vegetation communities would have lessened the amount of canopy protection and encourage wind erosion^55,56^. Similar floral adaptions were identified in the near-shore terrestrial vicinity of southwestern Africa, recording extensive grass-rich savannah and semi-arid vegetation since the very early Pleistocene^57^. More inland, vegetation cover in the southern Kalahari Desert was already reduced by 2 Ma, as suggested by the reconstructed C_3_-C_4_ floral assemblage^40,58^. These changes could have promoted sand transport deep into the heart of the continent throughout the early Pleistocene as recorded by sand preserved in the Wonderwerk Cave (Fig. 1a)^59^.

The distribution of unconsolidated sand would have been enhanced by the strengthening of winds due to the expansion of atmospheric convective cells. Such transitions in wind patterns were suggested to follow a major enlargement of ice sheets in the Southern Ocean that started at 3.3 Ma and culminated at 2.7 Ma^48,49^.

### The impact of sand on the environment

Mammalian and hominin evolution and their migration in eastern Africa were attributed to the interaction between tectonics and climate variability, especially where the effects of such drivers enabled the existence of lakes that formed supportive habitats for hominins^60^. This approach focuses on the accommodation space and precipitation regime that directly control the formation and extent of water bodies. However, in sand-dominated terrains, the effect of climate change on the interactions between ground and surface waters is highly transient, since seepage from waterbodies to groundwater takes place through most of its bed^61^. Moreover, damming of fluvial systems is common in erg-like landscapes^62^, thus limiting water transport into geomorphological depressions and the formation of perennial lakes within them.

The introduction of sand into southern Africa during the early Pleistocene due to tectonic and climatic events had therefore dramatically altered the environment by blanketing the landscape with massive sand deposits and encouraged biota to adapt accordingly. The ecological response to the changing environment is demonstrated by the speciation of near-shore flora and fauna at 2.7–2.2 Ma^57,63^ and stable isotopes in fossil eggshells that signify the ongoing desertification from the coast inland which sharply increased at ~2 Ma^35^. More inland, the emplacement of extensive sand deposits resulted in the desiccation of the Nossob-Molopo waterbody (Fig. 1a) that disappeared around 1 Ma, irreversibly altering the landscape for the hominins that occupied the area and was probably a vital turning point in their migration path to areas with higher water availability^21,30,40^.

## Methods

### ^26^Al and ^10^Be extraction

Amalgamated sand samples of ~500 gr were sieved in the lab and quartz was separated and purified from the 250–850 μm fraction to determine in-situ^26^Al and ^10^Be concentrations (Supplementary Data 2)^64^. The procedure involved sample leaching by aqua regia solution (3:1 of HCl:HNO_3_) at 50 °C, magnetic separation, and sequential HF + HNO_3_ etching. Be and Al spikes at known isotopic ratios were added prior to quartz digestion in HF, HClO_4_, and HNO_3_ mixture. Aliquots were extracted from the dissolved fraction for native Al measurements by ICP-MS at the Hebrew University of Jerusalem. Al^+3^ and Be^+2^ were extracted via ion-exchange chromatography and converted to oxides at 750 °C^65^.

Isotopic ratios of oxidized targets were measured by accelerator mass spectrometry at *Centre de Recherche et d’Enseignement de Géosciences de l’Environnement* (CEREGE), France. All ratios are corrected for AMS facility standards. AMS standard values for samples SAND13, SAND14, MM1, and MM3 are from^21^. AMS standard values for all other samples are: ^26^Al/^27^Al – 7.041*10^−12^; ^10^Be/^9^Be – 6.05*10^−12^. All ratios are corrected for procedural blanks. Procedural blank values range between 3.2*10^−15^ and 9*10^−16^ for ^26^Al/^27^Al and between 1.94*10^−15^ and 7.98*10^−15^ for ^10^Be/^9^Be. Each batch of samples was corrected by the value of the corresponding batch blank.

### Interpretation of ^26^Al and ^10^Be concentrations

Apparent burial ages were estimated for the detrital quartzose components in one carbonate-dominated sample and five overlying consolidated sandstone samples at the northeastern margin of the Koppieskraal Pan (Fig. 1; Supplementary Data 3). Two end-member, pre-burial, ^26^Al/^10^Be values were considered: (1) a ratio calculated individually for each sample through iteration (range between 5.03 and 5.79), assuming steady erosion^28^, and (2) a value estimated from the measured concentrations in current surface samples that overlie the buried samples. At the Koppieskraal Pan, we used the average value of two surface samples, i.e. 4.17, and for GAL6 and GAL3, we used the ratio of the NSB sample of 4.24. Because shallowly buried samples could be affected by long periods of stagnancy that are not represented in the datasets used to construct the vertical displacement rates, we constrained our estimation for the duration of eolian vitality of these samples (GAL3, GAL6) by calculating their burial age as well (Supplementary Data 3). The resulting apparent burial ages, calculated using the MATLAB implementation of^21^, set the plausible age range for the deposition of sediments. With the assumed pre-burial ratio, ^26^Al and ^10^Be concentrations were modeled as the product of build-up during exposure, followed by radionuclide decay and post-burial production by muons, assuming production rates of present-day sample depth^66^. Specific nuclide production was estimated using the time-varying scaling scheme^67^. Probability density function for each sample age was calculated via chi-squared inversion of a 2D Monte Carlo simulation which generates concentrations as the outcome of nuclide build-up and burial durations.

Eolian residence time for unconsolidated sand was modeled according to several possible scenarios constructed from thirty-six combinations of boundary conditions (Supplementary Data 4) using the Cosmolian program^27^. This program simulates the build-up of ^26^Al and ^10^Be through a 2D modeling of a dune generation scheme and compares simulated values to analytically measured values^22^. The model includes pre-eolian stage where cosmogenic nuclides are produced due to erosion at source areas, accounting for possible fluvial transport. Erosion rates of 3, 9, and 15 m Ma^−1^, typical of present African landforms, were used to model pre-eolian concentrations^65^. A higher value of 20 m Ma^−1^ was also assigned based on our calculations of paleo-erosion rates from ^10^Be concentrations of deeply buried sediments in the study area. During the eolian stage, the accumulation of ^26^Al and ^10^Be is calculated as a function of the retention time and the overburden at any depth, while stochastic vertical movement at 20 cm increments occurs. Three characteristic dune heights (5,10,20 m) were considered for the simulated amplitude of vertical displacement, and three datasets were used as vertical displacement rates for migrating eolian sand. These datasets are based on the conversion of luminescence and ^14^C ages of eolian and fluvial deposits in the study area (Supplementary Data 4) into retention times^22^. Using these datasets allows a realistic representation of chronological dune development within the limits of OSL dating, including dune stabilization. An additionally adjusted dataset was constructed to account for the possible bias stemming from oversampling young and shallow dune deposits by equating the probabilities of the shortest retention periods comprising 90% of the data to the 10% longer durations of the dataset^22^. Average production rates were calculated for the catchment area above each sampling area^68^, based on a digital elevation model with a resolution of 30 arc-seconds^69^.

The modeling results are summarized and presented as the most probable modeled time for convergence between measured and modeled ^26^Al and ^10^Be to occur and the percentage of such convergence events for each combination of boundary conditions (Supplementary Data 5). The results were analyzed by considering the peak of the kernel probability density estimation, which was calculated over a fixed interval of 10 kyr, using all the converged simulations of each sample so that the weighted uncertainty of the different scenarios is considered as well. The peak of the probability density estimation is considered as the most probable value representing the time passed since the sampled sand was introduced into the landscape.

The uncertainty associated with the dating, which is reflected by the width and the shape of the cumulative kernel density estimate was evaluated utilizing a half-width at half-height approach. Because the distributions of the model results are unimodal, and the uncertainty on the timing of the peak is minimal (mostly < 20 ky), the timing of the half-height was determined for each peak on both of its sides.

## Supplementary information

Description of Additional Supplementary Files

Supplementary Data 1

Supplementary Data 2

Supplementary Data 3

Supplementary Data 4

Supplementary Data 5

Supplementary Data 6

## Data Availability

All data generated or analyzed during this study are included in this published article (and its supplementary data and information files).

## Code availability

An installation file for the Cosmolian program is available as a Supplementary Information file. The readers may contact the authors for any future releases of Cosmolian, requests and comments.
