# Supplementary material for: Eolian chronology reveals causal links between tectonics, climate, and erg generation
Source: Nat Commun. 2022 Sep 29;13:5714. doi: 10.1038/s41467-022-33316-7 (PMC9522826; doi:10.1038/s41467-022-33316-7)
Supplement: Supplementary file 1 — Description of Additional Supplementary Files [file 41467_2022_33316_MOESM1_ESM.pdf]

File name: Supplementary Data 1

Description: Particle size distribution of surficial and buried Kalahari Sand

File name: Supplementary Data 2

Description: Al and Be cosmogenic nuclides data of surficial and buried Kalahari Sand

File name: Supplementary Data 3

Description: Samples field description and models results (burial age and eolian residence time)

File name: Supplementary Data 4

Description: Cosmolian input: geographical parameters, erosion rate at source rocks including fluvial transport, the dataset used for retention time during vertical displacement)

File name: Supplementary Data 5

Description: Cosmolian output: modeled time until convergence between analytical and simulated concentrations of 26Al and 10Be, the success rate of convergence to occur for each combination of parameters

File name: Supplementary Data 6

Description: Installation file for the Cosmolian program including an example spreadsheet.
